# Impact of DNA Prime/Protein Boost Vaccination against *Campylobacter jejuni* on Immune Responses and Gut Microbiota in Chickens

**DOI:** 10.3390/vaccines10060981

**Published:** 2022-06-20

**Authors:** Noémie Gloanec, Daniel Dory, Ségolène Quesne, Véronique Béven, Typhaine Poezevara, Alassane Keita, Marianne Chemaly, Muriel Guyard-Nicodème

**Affiliations:** 1 GVB–Viral Genetics and Biosafety Unit, French Agency for Food, Environmental and Occupational Health and Safety (ANSES), 22440 Ploufragan, France; noemie.gloanec@anses.fr (N.G.); veronique.beven@anses.fr (V.B.); 2 HQPAP–Unit of Hygiene and Quality of Poultry and Pork Products, French Agency for Food, Environmental and Occupational Health and Safety (ANSES), 22440 Ploufragan, France; segolene.quesne@anses.fr (S.Q.); typhaine.poezevara@anses.fr (T.P.); marianne.chemaly@anses.fr (M.C.); muriel.guyard@anses.fr (M.G.-N.); 3 UFR of Life Sciences Environment, University of Rennes 1, 35700 Rennes, France; 4 SELEAC–Avian Breeding and Experimental Department, French Agency for Food, Environmental and Occupational Health and Safety (ANSES), 22440 Ploufragan, France; alassane.keita@anses.fr

**Keywords:** flagellin antigen, poultry, innate immunity, systemic and mucosal immune response, *Campylobacter jejuni* caecal colonisation, caecal microbiota composition

## Abstract

*Campylobacteriosis* is reported to be the leading zoonosis in Europe, and poultry is the main reservoir of *Campylobacter.* Despite all the efforts made, there is still no efficient vaccine to fight this bacterium directly in poultry. Recent studies have reported interactions between the chicken immune system and gut microbiota in response to *Campylobacter* colonisation. The present study was designed to analyse in more depth the immune responses and caecal microbiota following vaccination with a DNA prime/protein boost flagellin-based vaccine that induces some protection in specific-pathogen-free White Leghorn chickens, as shown previously. These data may help to improve future vaccination protocols against *Campylobacter* in poultry. Here a vaccinated and a placebo group were challenged by *C. jejuni* at the age of 19 days. A partial reduction in *Campylobacter* loads was observed in the vaccinated group. This was accompanied by the production of specific systemic and mucosal antibodies. Transient relatively higher levels of Interleukin-10 and antimicrobial peptide avian β-defensin 10 gene expressions were observed in the vaccinated and placebo groups respectively. The analysis of caecal microbiota revealed the vaccination’s impact on its structure and composition. Specifically, levels of operational taxonomic units classified as *Ruminococcaceae* and *Bacillaceae* increased on day 40.

## 1. Introduction

*Campylobacter*iosis is the most frequently reported zoonosis in the European Union, with 220,682 human cases in 2019 [1]. The causative agent is *Campylobacter*, a microaerophilic gram-negative bacterium. *Campylobacter jejuni* is the most frequently reported species. *Campylobacter* is generally responsible for an acute gastrointestinal illness [2] and is also associated with extra-gastrointestinal infections like bacteraemia, meningitis and urinary infections. It is also considered to be a major trigger for a severe demyelinating neuropathy known as Guillain–Barré syndrome [3]. Poultry is the main reservoir, and the majority of infections are attributed to the consumption and handling of poultry meat products contaminated with *C. jejuni* [1]. The predominant site of *C. jejuni* colonisation in the chicken gut is the caecum, which can generally contain about 8 log_10_ colony-forming units (CFU)/g), a high colonisation level correlated with contamination during slaughter and carcass processing [4,5]. As *Campylobacter*iosis is a major public health issue with important economic consequences, increased surveillance and control measures at primary production level are needed to reduce the risk to consumers. It is estimated that a reduction by 2 log_10_ or 3 log_10_ CFU/g of *Campylobacter* levels in broiler caeca would reduce the relative risk of human *Campylobacter*iosis by 42% or 58% respectively [6]. Consequently, the European Union implemented a microbiological criteria plan for broiler chicken carcasses with a limit of 3 log_10_ CFU/g on neck skin [7]. Vaccinating poultry is considered to be one of the potential measures to reduce *Campylobacter* carriage in chickens [8,9,10,11,12]. However, no effective vaccine is currently available on the market despite the many publications on the subject. Furthermore, studies seeking to depict the mechanism behind *Campylobacter* colonisation in poultry demonstrate the involvement of (1) maternally-derived immunity; (2) innate immune responses with the involvement of Toll-like receptors and β-defensins which are antimicrobial peptides (AMPs); (3) adaptive immune responses with the production of antibodies or cytokines/chemokines; as well as changes in (4) gut microbiota structure and composition [13,14,15,16,17,18,19,20]. The immune system detects infection of the gastrointestinal tract by pathogens and responds by several interconnected pathways involving the innate and adaptive immune systems and allows a cooperation and a retroaction between these two systems. The gut microbiota plays an important role through their effects on nutrient exchange, gut morphology, regulation of mucin production, modulation of immune system, detoxification, protection against pathogens and health, growth and performance of chickens [21]. The caecum constitutes the organ with the highest taxonomic abundance and diversity mainly, because feed circulates in the caeca for the longest period (from 12 to 20 h) in the chicken digestive tract [22]. The chicken caecal microbiota, characterised by modern sequencing approaches, is constituted of three main bacterial phyla: *Firmicutes*, *Bacteroidetes* and *Proteobacteria* [23]. In the chicken gut *Campylobacter* interacts with other microorganisms and plays a role in structure and composition of the microbiota [19,24,25,26,27], despite this, the bacterium is cited as a commensal inhabitant of the gut microbiota in chicken [28]. Both the immune system and the microbiota should therefore be taken into consideration during anti-*Campylobacter* vaccine development studies.

In a previous study [10], it was shown that the inoculation of a flagellin-based DNA prime/protein boost vaccine regimen against *Campylobacter* resulted in a dramatic reduction in caecal *Campylobacter* loads in specific-pathogen-free (SPF) Leghorn chickens at slaughter (dropping from around 8 log_10_ CFU/g to below the detection limit of 2 log_10_ CFU/g). It is therefore a suitable model for evaluating the immune and microbiota parameters involved in protective immunity. These data may be very useful for identifying novel strategies to improve vaccine efficacy against *Campylobacter*. However, this first trial neither studied immune responses in depth nor evaluated the role of caecal microbiota.

The present work was therefore designed to study these parameters more closely through another in vivo trial with the same vaccine regimen and chicken breed. The following parameters were measured at different time points starting from the day of *Campylobacter* challenge (19-day-old chickens) to the end of rearing (40-day-old chickens): caecal *Campylobacter* loads, production of specific serum IgY and bile IgA antibodies reflecting, respectively, the systemic and mucosal humoral immune responses, a panel of key caecal cytokines, chemokines and antimicrobial peptide gene expressions and microbiota composition.

In this study, the flagellin-based DNA prime/protein boost vaccine regimen reduced *Campylobacter* loads (mean reduction of 1.3 log_10_ on day 40) in the caecum and stimulated the production of specific antibodies in sera (IgY) and bile (IgA) throughout the same period (D19-D40) as well as a transient gene over-expression of Interleukin-10 (IL-10) in the caecum on day 28. In the placebo group, there was a gene over-expression of antimicrobial peptide avian β-defensins 10 (AvBD10) on day 40. Moreover, vaccination was observed to induce changes in the microbiota’s structure and composition at different time points, including, in particular, an increase in *Ruminococcaceae* and *Bacillaceae* families in the vaccinated group at the age of 40 days.

## 2. Materials and Methods

### 2.1. Production of DNA Vaccine

The plasmid encoding *C. jejuni* flagellin (pcDNA3-flagellin A (FlaA)) and the placebo, pcDNA3, were produced and characterised as described previously [10].

For each chicken, 150 µg of pcDNA3-FlaA (or pcDNA3 for the placebo group) was mixed with 25 µg of unmethylated CpG ODN2007 (TCGTCGTTGTCGTTTTGTCGTT, with phosphorothioate backbone) (Sigma-Aldrich, Saint-Quentin-Fallavier, France) and then stored at −20 °C until vaccination.

### 2.2. Production of the Recombinant Flagellin (recFlaA) Protein Vaccine

Recombinant *C. jejuni* flagellin A (recFlaA) was produced previously [10]. For each chicken, 100 µg of recFlaA protein (or phosphate buffer saline (PBS) for the placebo group) was emulsified in MONTANIDE^TM^ ISA 71 VG (30/70, p/p) (Seppic, La Garenne-Colombes, France) the day before vaccination and stored at 4 °C.

### 2.3. Campylobacter Strain and Growth

The *C. jejuni* C97Anses640 strain, isolated from a poultry product and belonging to the ST−45 (sequence type) complex, was used for the in vivo oral challenge. Bacteria from frozen stock (−70 °C) were plated twice on selective modified charcoal cefoperazone deoxycholate agar (mCCDA) (Thermo Fisher Diagnostics, Dardilly, France) for 48 h at 41.5 °C under microaerobic conditions (85% N_2_, 10% CO_2_ and 5% O_2_), then inoculated twice in Brucella broth (Becton Dickinson, Le Pont-de-Claix, France) for 24 h under the same incubation conditions. The bacterial suspension was diluted to 5 log_10_ CFU/mL in tryptone salt broth (BioMérieux, Bruz, France) and the concentration of bacterial suspension was confirmed by plating the inoculum on mCCDA plates in 10-fold dilution series.

### 2.4. Avian Vaccine Experiment

A total of 72 day-of-hatch SPF White Leghorn layer chicks were provided by ANSES facilities (certification no. D-22–745-1). At the beginning of the experiment, the absence of *Campylobacter* spp. was confirmed in the experiment rooms (including the feeding and drinking systems) and in five chicks. The remaining birds were randomly divided into a vaccinated (*n* = 35) and a placebo (*n* = 32) group and were kept in floor pens (1.85 × 1.85 m^2^) in separate rooms.

The diets during the experiment were fairly standard diets for poultry, formulated and manufactured by a commercial feed meal company. The two groups received a starter-grower diet from days 1 to 19 then a grower-finisher diet until day 40 (D40).

**Figure 1:**
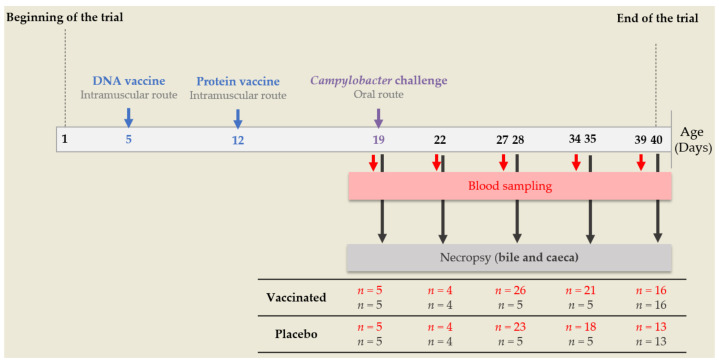
Main steps of the experimental procedure. Each day, the number of chickens (*n*) used for blood sampling and necropsy is represented in red or brown respectively.

DNA prime and protein boost vaccinations were performed on days 5 and 12 respectively using 26 G needles for intramuscular injections. On D19, all the chickens were orally challenged with 10^5^ CFU of *C. jejuni* C97Anses640 isolated at the ANSES laboratory in Ploufragan from a poultry product, and already used in previous trials [9,29]. On D19, D22, D27, D34 and D39, blood samples were taken from the occipital sinus. The sera were recovered after coagulation and centrifugation (2000× *g*, 10 min, room temperature) and stored at −20 °C until determination of the systemic immune response. On D19, D22, D28, D35 and D40, 4 to 16 birds per group were euthanised (electronarcosis followed by bleeding). Bile samples were taken and stored at −20 °C until evaluation of the mucosal immune response. All caecal contents were collected for *Campylobacter* spp. enumeration and microbiota analysis. A portion (around 0.5 cm long) of caecal wall was immediately placed in 1 mL of RNAlater^TM^ (Invitrogen), incubated for one day at 4 °C then stored at −80 °C until gene expression assessment. On days 5, 19 and on each slaughter day, each bird was individually weighed. Birds were observed daily to ensure that no adverse reactions occurred. The main steps of the experimental design are summarised in Figure 1.

### 2.5. Campylobacter Caecal Enumeration

*Campylobacter* enumerations were assessed in caecal contents after direct plating according to the decimal dilution method. Caeca were homogenised then serially diluted 1:10 (*w*/*v*) up to dilution 10^−6^ in tryptone salt broth (Biomerieux, Craponne, France) and plated on mCCDA using an automatic easySpiral Dilute ^®^ plater (Interscience, Saint-Nom-la-Bretèche, France). Typical *Campylobacter* colonies were enumerated on plates after incubation for 48 h at 41.5 °C under microaerophilic conditions (85% N_2_, 10% CO_2_ and 5% O_2_) and converted to log_10_ CFU/g. The detection limit for enumeration of *Campylobacter* was 2 log_10_ CFU/g. A sample of one mL of the first dilution (10^−1^) of caecal content was centrifuged (10,000× *g*, 10 min) and the pellet was stored at −70 °C until microbiota analysis.

### 2.6. Serum (IgY) and Bile (IgA) Anti-Flagellin Antibodies by Specific ELISAs

The levels of antibodies against flagellin protein in serum and bile were measured by ELISA by adapting the method of [10], in which the plates were coated with the purified flagellin instead of *Campylobacter* soluble proteins. Briefly, 96-well Maxisorp^®^ plates (Nunc) coated with 100 µL of 2 µg/mL of purified flagellin protein per well were successively incubated with 1:4800 diluted serum or 1:100 diluted bile, followed by 1:35,000 diluted goat anti-chicken IgY- horseradish peroxidase (HRP) antibodies (Abcam, Paris, France) or 1:5000 diluted goat anti-chicken IgA-HRP respectively. After incubation with *o*-phenylenediamine dihydrochloride substrate in citrate buffer containing hydrogen peroxide, the reaction was stopped by adding 1 M H_2_SO_4_. The optical densities (ODs) were measured at 490 nm using a spectrophotometer (Infinite 200 PRO Nanoquant, Tecan, Lyon, France). Each plate contained one serum internal control and one bile internal control to allow standardisation between experiments and each sample was measured in duplicate.

### 2.7. Relative Cytokine and Chemokine Expressions Determined by RT-qPCR

#### 2.7.1. RNA Extraction

RNA was extracted from caecal tissues using Agencourt^®^ RNAdvance^TM^ Tissue kit (Beckman coulter, Brea, CA, USA) with magnetic beads according to the manufacturer’s protocol with the following modifications. Tissue samples were homogenised in a lysis buffer with 2.8-mm ceramic beads (MO BIO Laboratories Inc., Carlsbad, CA, USA) using a ball mill (Retsch, Haan, Germany). The supernatant was collected (400 µL) after centrifugation at 4 °C for 10 min prior to subsequent purification as described in the manufacturer’s protocol. RNA was eluted in RNAse-free water then processed with the Turbo DNA-free™ kit (Thermofisher Scientific, Vilnius, Lithuania), quantified using the Qubit RNA high sensitivity assay kit (Life Technologies Corporation, Eugene, OR, USA) with a Qubit 2.0 fluorometer (Life Technologies, Saint-Aubin, France) and stored at −80 °C. Some samples were checked for total RNA quality and integrity by capillary electrophoresis using an Agilent fragment analyser system.

#### 2.7.2. Reverse Transcription of Total RNA

The cDNAs were obtained from 320 ng of total RNAs using the High-capacity cDNA Reverse Transcription kit (Applied Biosystems, Villebon sur Yvette, France) according to the manufacturer’s instructions, then stored at −20 °C.

#### 2.7.3. qPCR

The relative levels of cytokine, chemokine and AMP RNA were determined by qPCR using the 7500 real-time PCR system (Applied Biosystems, Villebon sur Yvette, France). A quantitative PCR reaction was performed with a cDNA template retrotranscribed from 4 ng of total RNA in duplicate using SYBR Green Master mix (Applied Biosystems, ThermoFisher Scientific, Villebon sur Yvette, France) and the following programme: 95 °C/10 min followed by 40 amplification cycles (95 °C/15 s, 60 °C/1 min). The primers used for a qPCR of β-actin, interferon (IFN)-γ, interleukin (IL)8-like (L)1, IL8L2, IL-1β, IL-4, IL-10, IL-17A, antimicrobial peptide avian β-defensins 10 (AvBD10) and AvBD12 are presented in Table 1. Specific primers for IL-10 were designed using Primer Express^®^ (Applied Biosystems, Villebon sur Yvette, France) software. Each qPCR reaction performed in 20 µL of reaction mix consisted of 1X SYBR Green Master mix (Applied Biosystems, ThermoFisher Scientific, Villebon sur Yvette, France), 300 mM of each primer, RNAse-free water and the retrotranscribed cDNA. The absence of any genomic DNA contamination was checked by qPCR on the total RNA. The relative amount of target gene expression was determined by the 2^−ΔΔCt^ method [30] with β-actin as the reference gene [31]. PCR efficiency, measured using the slope of a standard curve, ranged from 90 to 110%. Statistical tests were performed using duplicates of the 2^−ΔCt^ data for each gene in the vaccine and placebo groups [19].

### 2.8. Statistical Analyses

R software (version 4.0.3) was used for statistical analysis. Student’s parametric test was used when the normality and homogeneity criteria of the variances were validated (checked by the Shapiro–Wilk normality test and Bartlett’s test respectively); otherwise, the non-parametric Mann–Whitney test was used. A *p*-value lower or equal to ≤0.05 (*p* ≤ 0.05) was considered statistically significant.

### 2.9. DNA Extraction and PCR Amplification of 16S rRNA Gene Sequences and Microbiota Diversity Analysis

#### 2.9.1. DNA Extraction

Bacterial DNA was isolated from caecal pellets using the NucleoMag Tissue Kit (Macherey-Nagel, Hoerdt, France). Briefly, caecal pellets were resuspended in 500 µL of T1 lysis buffer and were mechanically lysed by adding one stainless-steel 5 mm bead (Qiagen, Courtaboeuf, France) and using the Star Beater (3 min–30 Hz) (VWR, Fontenay-sous-Bois, France). Chemical lysis was performed by incubation (30 min–70 °C) with 25 µL of Proteinase K (NucleoMagTissue kit, Hoerdt, France). After centrifugation (2 min–13,000× *g*), 225 µL of supernatant was transferred into a standard 96-well plate and the DNA was extracted with a KingFisher Duo Prime instrument (Thermofisher Scientific, Illkirch-Graffenstaden, France). DNA extraction of samples from the different groups was randomly performed and a negative extraction control was used for each plate. DNA concentrations were determined using the Qubit dsDNA High Sensitivity (HS) Assay Kit (Thermofisher Scientific, Illkirch-Graffenstaden, France) and a Qubit 2.0 Fluorometer (Life Technologies, Saint-Aubin, France) and were then stored at −20 °C until use.

#### 2.9.2. Sequencing of the V3/V4 Variable Region of the 16S Ribosomal Genes

Genomic DNA from all samples was PCR-amplified using a primer set covering the V3-V4 variable regions of the 16S rDNA gene (forward primer: 5′-TCGTCGGCAGCGTCAGATGTGTATAAGAGACAG; reverse primer: 5′ GTCTCGTGGGCTCGGAGATGTGTATAAGAGACAG) and the expected amplicon size is approximately 460 bp (15044223 Rev B adapted). The 2 × 300 bp paired-end sequencing of the amplicons was performed using an Illumina MiSeq sequencer with the Illumina MiSeq reagent kit 600 version 3, according to the Illumina 16S metagenomic library preparation protocol (15044223 Rev B adapted) and MiSeq system denature and dilute libraries guide (15039740–3 December 2017 adapted).

#### 2.9.3. Sequence Analyses

Sequences were processed using FROGS (Version 3.2.3 + galaxy2) [36], a galaxy-supported pipeline. Briefly, paired-end reads were merged using VSEARCH, and sequences were cleaned by removing those with ambiguous bases, those of an unexpected length (<380 or >500 nucleotides), or those without a primer sequence at both 3′- and 5′-ends (no mismatch allowed) before dereplication. After the sequences were clustered into operational taxonomic units (OTUs) using SWARM, and following FROGS guidelines, chimeras were removed using VSEARCH combined with a cross-sample validation step. The OTUs were then filtered according to their size (OTUs with an abundance below 5 × 10^−5^ were removed) and the BLAST algorithm was used for taxonomic assignment against the SILVA 16S database (version 132 filtered at a pintail score of 80).

#### 2.9.4. Statistical Analyses

Descriptive statistical analyses on the diversity and structure of caecal microbiota were performed using the phyloseq R package implemented in FROGS. The richness of a sample is defined by the number of OTUs (observed richness). Chao1 richness index estimates total richness (observed richness and the unknown number of species present in the community but not observed). Shannon and InvSimpson are used to describe diversity in samples and take into account richness and evenness (Table S1). The effect of the vaccination on these indices was investigated using ANOVA. We also investigated the impact of vaccination on microbiota diversity and structure. A weighted UniFrac (wUniFrac) distance matrix, which takes into account the relative abundance of OTUs shared between samples was calculated after data rarefaction and plotted using multidimensional scaling (MDS) to investigate the structure of the bacterial community. An ADONIS pairwise test was used to check significance. To identify and visualise whether taxa with differential abundance were statistically different between vaccinated and placebo groups, the linear discriminant analysis (LDA) effect size (LEfSe) method was used, and only OTUs of family and genera with an LDA score over 2 were reported on D40. LEfSe analysis was performed with all taxonomic ranks but only genera and families are represented graphically.

## 3. Results

### 3.1. Body Weight

The in vivo trial was performed on SPF Leghorn chickens to assess the effect of vaccination with *C. jejuni* flagellin on caecal *C. jejuni* loads. Chickens were injected by a DNA prime/protein boost flagellin-based vaccine or a placebo at days 5 (DNA) and 12 (protein) as described above. The chickens were then orally challenged with *Campylobacter* on day 19.

Results showed that the vaccination did not affect chicken growth, as there was no difference in mean body weight between the placebo and vaccinated groups (*p* > 0.05) during the whole rearing period (Table 2). No adverse reaction was observed in either of the two groups.

### 3.2. Campylobacter Caecal Enumeration

**Figure 2:**
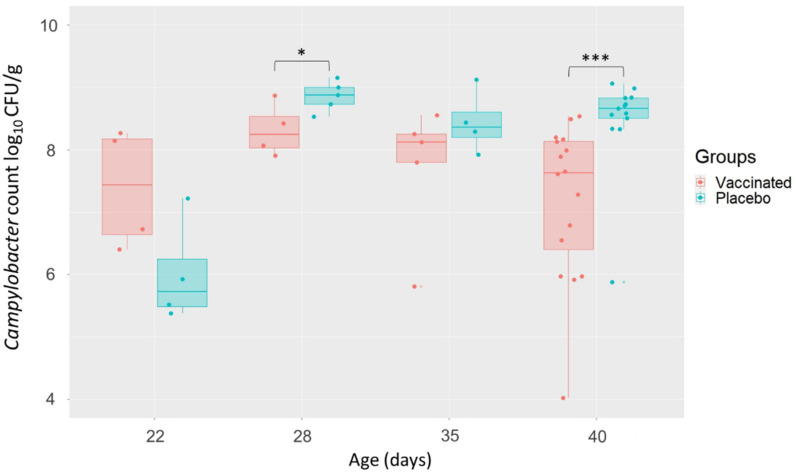
Effect of vaccination on *Campylobacter* colonisation of chicken caeca according to the age of the chickens (corresponding to the experiment days). Significant differences between the two groups are indicated by asterisks (* *p* < 0.05, *** *p* < 0.001; Wilcoxon rank sum test).

*Campylobacter* enumerations were assessed from caecal contents at different time points post inoculation. The results are presented in Figure 2. On D22, three days after the challenge, *Campylobacter* colonisation was tested. Both the vaccinated and placebo groups were colonised by high levels of *Campylobacter* (from 5.4 to 8.3 log_10_ CFU/g) of caecal content. A significant reduction (*p* < 0.05) in caecal *Campylobacter* load was observed in the vaccinated group compared with the placebo group on D28 (mean). A significant reduction (*p* < 0.001) was also observed at the end of rearing on D40 with a mean difference of 1.3 log_10_ (7.2 log_10_ vs. 8.5 log_10_) between the two groups despite high inter-individual variability in the vaccinated group.

### 3.3. Serum (IgY) and Mucosal (IgA) Anti-Flagellin Antibody Levels

**Figure 3:**
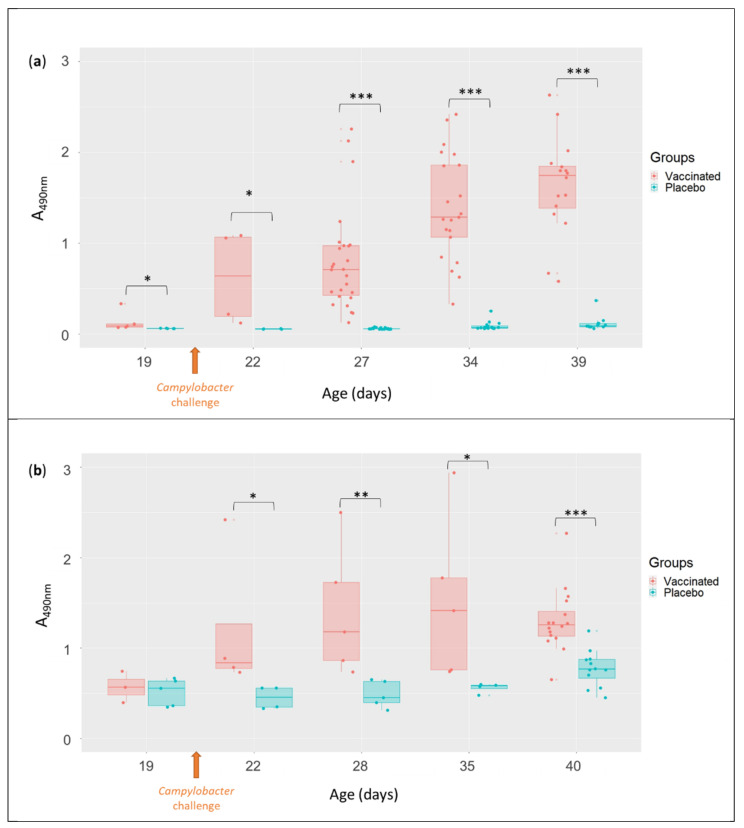
Anti-flagellin antibody levels after vaccination and challenge. (**a**) Levels of anti-flagellin IgY antibodies in serum. (**b**) Levels of anti-flagellin IgA antibodies in bile. Significant differences between the two groups are indicated by asterisks (* *p* < 0.05, ** *p* < 0.01, *** *p* < 0.001; Wilcoxon rank sum test).

The levels of antibodies against flagellin protein in serum (IgY type), which reflect the systemic humoral immune response, and in bile (IgA type), which reflect the mucosal humoral immune response, were quantified by ELISA. The levels of IgY in serum were significantly higher in the vaccinated group than in the placebo group from D19 to D40 (Figure 3a). Similarly, the levels of IgA in bile from D22 to D40 were significantly higher in the vaccinated group (Figure 3b).

In both cases, increases in antibody levels and high inter-individual variabilities were observed over point in the vaccinated group (Figure 3a,b) but not in the placebo group. IgY levels were individually measured on the same animals at different time points from D27 to D39. This monitoring showed that the level of IgY increased from D27 to D39 or reached a plateau from D34 for most of the vaccinated animals (data not shown).

### 3.4. Relative Expressions of Cytokines/Chemokines and Antimicrobial Peptides

**Figure 4:**
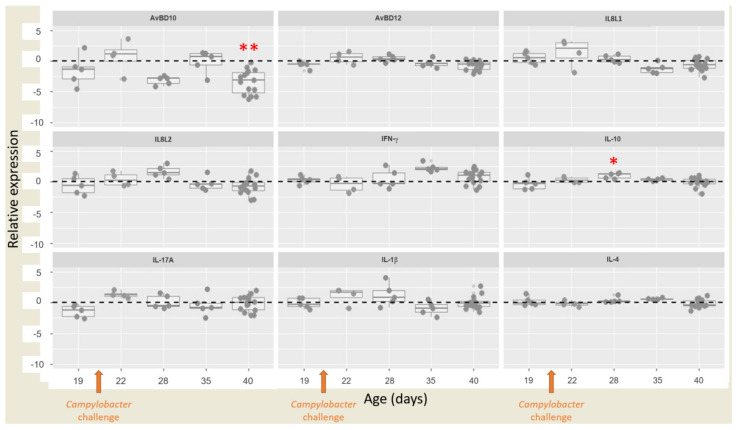
Relative gene expressions of cytokines, chemokines and AMPs in caecum. Relative gene expression represents log_2_ ratio vaccinated/placebo. Values > 0 (above the dotted black line) represent relative over-expressions of cytokine, chemokine or AMP genes in the caecum of vaccinated chickens compared with the placebo group while values < 0 (below the dotted black line) represent relative sub-expressions of cytokine, chemokine or AMP genes in the caecum of vaccinated chickens compared with the placebo group. Student’s parametric test was used when the normality and homogeneity criteria of the variances were validated (checked by the Shapiro–Wilk normality test and Bartlett’s test respectively); otherwise, the non-parametric Mann–Whitney test was used. Significant differences between 2^−ΔCt^ values of the vaccinated and placebo groups are indicated by asterisks in red (* *p* < 0.05, ** *p* < 0.01) for the expression of each gene at the corresponding time points. The genes evaluated were interferon (IFN)-γ, interleukin (IL)8-like(L)1, IL8L2, IL-1β, IL-4, IL-10, IL-17A, antimicrobial peptide avian β-defensins 10 (AvBD10) and AvBD12.

RT-qPCR was performed on caecal tissue from D19 to D40 to assess the relative expression of cytokines, chemokines and AMP genes that were previously shown to play a role in response to *Campylobacter* infection. AvBD10 and AvBD12 are AMPs involved in innate immune response [37]. As previously described, IFN-γ and IL-4 are markers of Th1 and Th2 pathways respectively. The Th17 pathway is represented by chemokines IL8L1 and IL8L2 and cytokines IL-1β and IL-17A whereas IL-10 is related to the Treg (regulatory T) cell pathway [38]. Relative gene expression between the vaccinated and placebo groups were calculated according to Connerton [19]. Out of the nine targets considered, only two differed in expression between the two groups (Figure 4). IL-10 characteristic of the anti-inflammatory Treg pathway was over-expressed on day 28 in the vaccinated group (*p* < 0.05). On the contrary, AvBD10 was under-expressed in vaccinated chickens on D40 compared with the placebo group (*p*< 0.01). Furthermore, after the *Campylobacter* challenge, a pro-inflammatory activity characterised by a slight over-expression of IL8L1, IL8L2, IL-17A and IL-1β from D22 to D28 and by IFN-γ from D22 to D35 appeared to be observed in the vaccinated group, but this relative over-expression was not significant *p* > 0.05) (Figure 4).

### 3.5. Caecal Microbiota Analyses

To assess the impact of vaccination on caecal microbiota, the caecal content of the chickens before the *Campylobacter* challenge on D19 (five chickens/group) and after colonisation on D22 (four chickens/group) and D40 (five randomly selected chickens/group) were analysed using 16S metabarcoding.

The number of sequences and OTUs obtained are indicated in Table 3.

**Figure 5:**
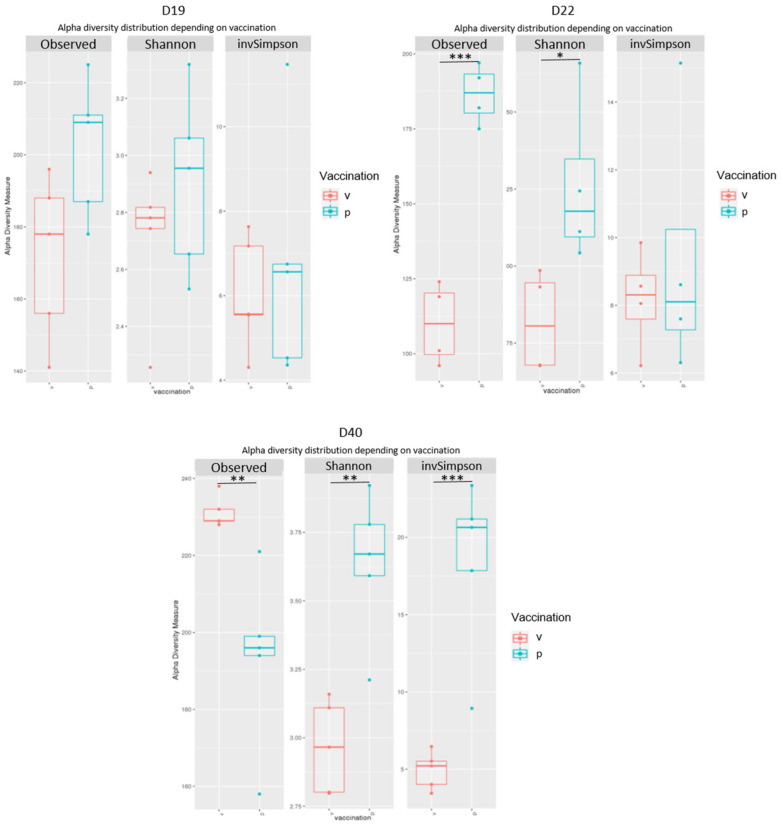
Richness and alpha diversity indices for chicken’s caecal microbiota on D19, D22 and D40. Significant differences between the vaccinated (v) group in red and placebo (p) group in blue are indicated by asterisks (* *p* < 0.05, ** *p* < 0.01, *** *p* < 0.001).

Number of OTUs, inverse Simpson and Shannon indices were determined to analyse richness and alpha diversity within samples (Figure 5). No significant differences in richness and diversity (for either index) were observed between the vaccinated and placebo groups on D19 (*p* > 0.05) before the *Campylobacter* challenge. On day 22, the richness (number of observed OTUs) was significantly lower in the vaccinated group (*p* < 0.001) than in the placebo group, but a shift was observed on day 40 and richness was significantly higher in the vaccinated group (*p* < 0.01). On D40 lower Shannon and inverse Simpson indices were observed in the vaccinated group (*p* < 0.01 and *p* < 0.001 respectively) reflecting a lower diversity. These results suggest that on D40 the vaccinated group contained more species (higher richness) but these species were less evenly distributed. Consequently, it appears that communities are dominated by fewer abundant taxa in the vaccinated group than in the placebo group. Table S1 shows the alpha diversity index values for each caecal sample.

**Figure 6:**
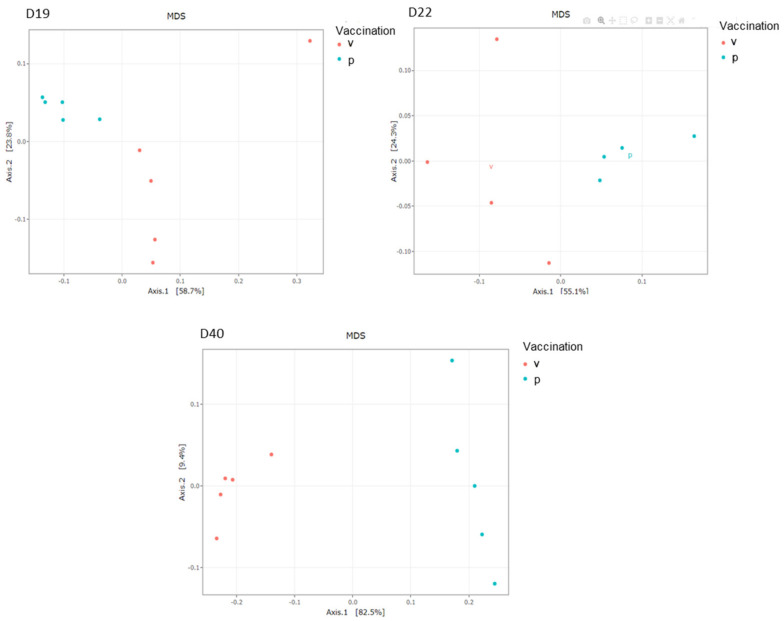
Representation of beta diversity of the chicken caecal microbiota on D19, D22 and D40. MDS based on weighted UniFrac distance. The vaccinated (v) group is in red and the placebo (p) group in blue.

A multi-dimensional scaling (MDS) of weighted UniFrac (wUniFrac) distances was performed to reveal differences in microbial population structures among samples depending on vaccination (Figure 6). A multivariate ANOVA (performed with Adonis) revealed a significant difference in microbial population structure between vaccinated and placebo groups regarding vaccination on D19 (*p* < 0.01), D22 (*p <* 0.05) and D40 (*p <* 0.01). The vaccination explained about 43%, 42% and 80% of the total variance on D19, D22 and D40 respectively. The samples were increasingly segregated over time according to their experimental group from D19 to D40 (Figure 6). Moreover, segregation of the samples using the wUniFrac distance suggests that the most abundant OTUs in the vaccinated group are phylogenetically distant from the OTUs in the placebo group.

**Figure 7:**
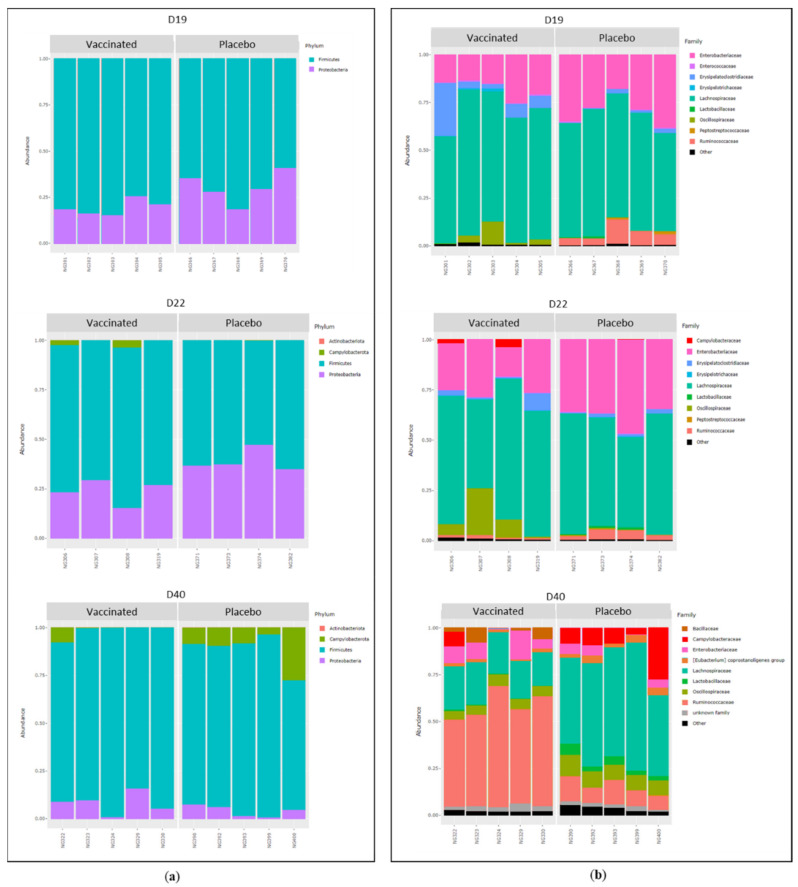
Taxonomic composition represented by relative abundance of bacterial communities from the caecal microbiota of chickens from the vaccinated (v) and placebo (p) groups on D19, D22 and D40. (**a**) Relative abundance of phyla identified in caecal microbiota represented by a bar for each sample. (**b**) Relative abundance of the nine main families represented by a bar for each sample.

The taxonomic composition of the chicken caecal microbiota is represented in Figure 7.

The results revealed only the presence of the *Firmicutes* phylum followed by *Proteobacteria* on D19. On D22 and D40, four phyla were identified: *Firmicutes* and *Proteobacteria* were predominant, followed by *Campylobacterota* then *Actinobacteriota* (Figure 7a). On D19 and D22, two bacterial families—*Lachnospiraceae* and *Enterobacteriaceae—*predominated in the samples. On D40, a higher number of bacterial families were present in the samples and a marked effect according to experimental group was observed (Figure 7b). Indeed, *Ruminococcaceae* predominated in the bacterial populations of the vaccinated group, whereas *Lachnospiraceae* predominated in the placebo group. Furthermore, the relative abundance of the *Bacillaceae* family was higher in the vaccinated group whereas the relative abundance of the *Lactobacillaecae* family was higher in the placebo group. Moreover, the relative abundance of *Campylobacteraceae* was higher in most of the placebo group birds.

**Figure 8:**
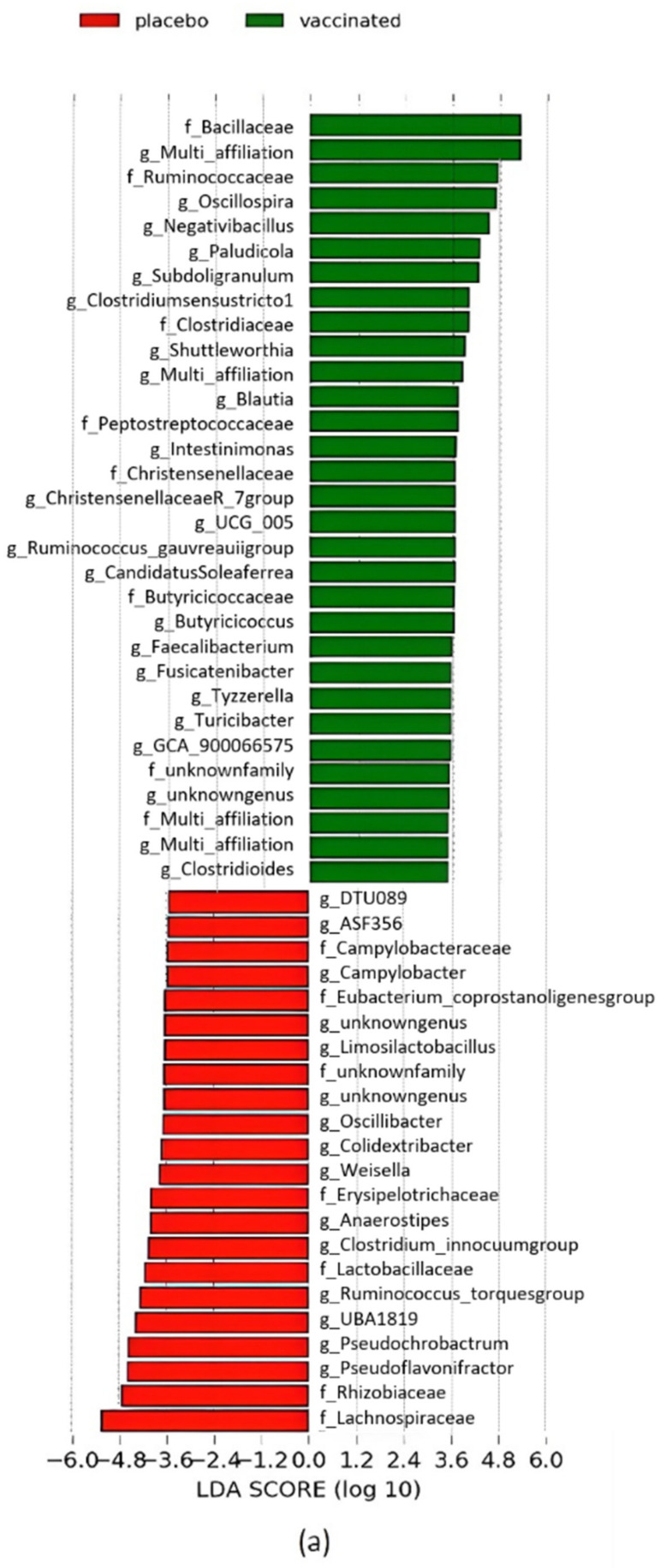

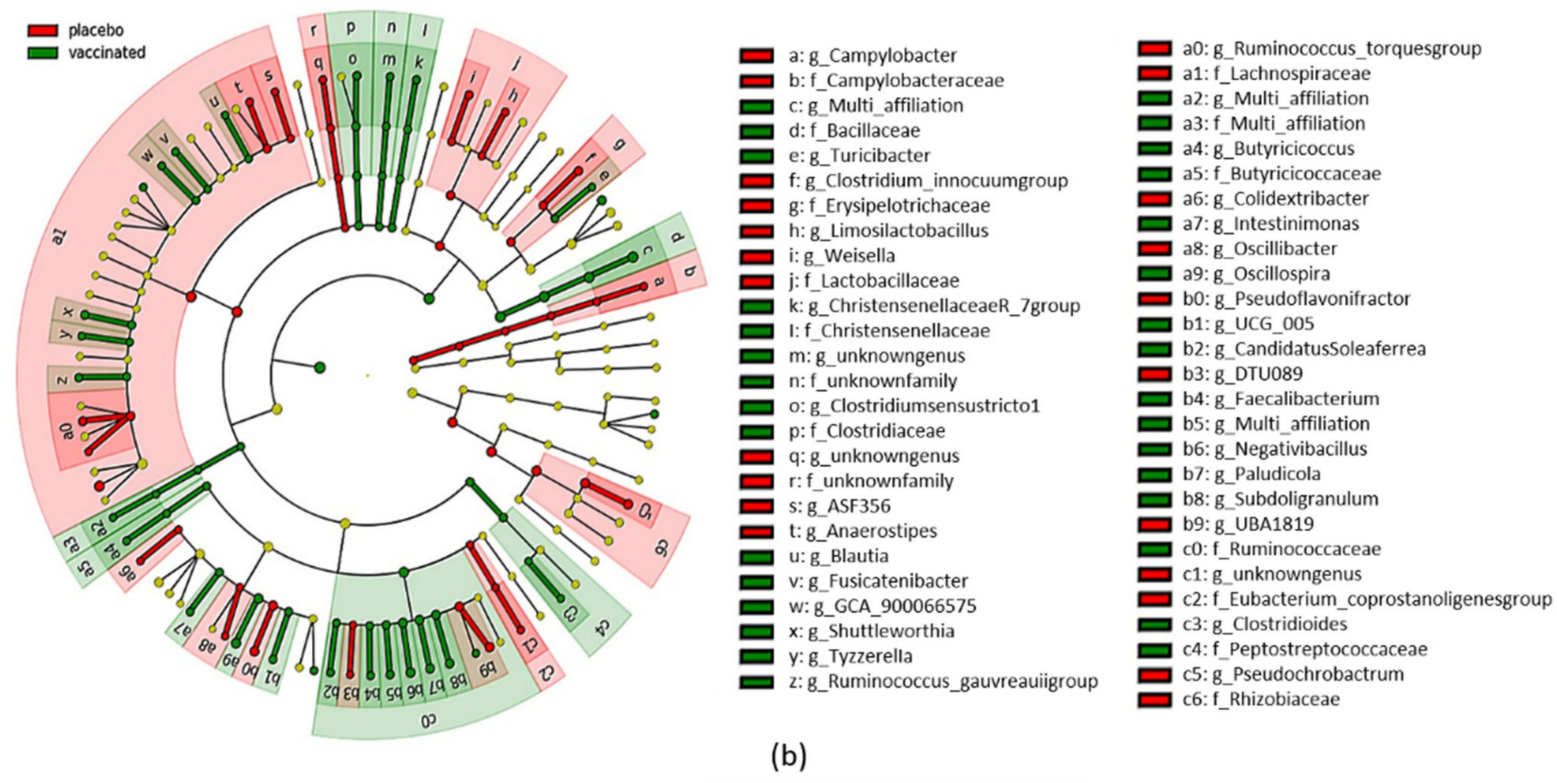
Differential abundances for members of the caecal microbial communities from the vaccinated and placebo groups on D40, identified by linear discrimination analysis coupled with effect size (LefSE). (**a**) Histogram of the LDA scores computed for taxa with significantly different relative abundance in the vaccinated group in green and placebo group in red. Only taxa belonging to family and genera with an LDA threshold value > two are reported. (**b**) Cladogram representing LefSE results of the identified taxa according to their phylogenetic characteristics. The green circles represent those of greater abundance in the vaccinated group, and red circles those in the placebo group. Yellow indicates non-significant differences between the two groups. The diameters of the circles are proportional to the taxon’s abundance. This representation highlights the presence of the differentially abundant taxonomic levels (f_ family, g_genera) as concentric arcs.

In the light of these results, a LEfSe analysis was performed to identify OTUs with a statistically different relative abundance between the vaccinated and placebo groups on D40 (Figure 8). Figure 8 presents only bacterial families and genera with an LDA score over two. The vaccinated group contained in particular (*p* < 0.05) *Bacillaceae*, *Ruminococcaceae*, *Clostridiaceae*, *Peptostreptococcaceae*, *Christensenellaceae* and *Butyricicoccaceae* families whereas the placebo group contained in particular (*p* < 0.05) *Campylobacteraceae*, *Eubacterium_coprostanoligenesgroup*, *Erysipelotrichaceae*, *Lactobacillaceae*, *Rhizobiaceae*, *Lachnospiraceae* and *Campylobacteriaceae* families on D40. Some genera, including *Oscillospira*, *Negativibacillus*, *Blautia* and *Faecalibacterium,* were significantly (*p* < 0.05) more abundant in the vaccinated group whereas others, including *Pseudoflavonifractor*, *Ruminococcus_torquesgroup*, *Colidextribacter* and *Oscillobacter*, were significantly (*p* < 0.05) more abundant in the placebo group on D40. Table S2 shows the complete taxonomic composition of caecal microbiota in each sample, including the relative abundance of OTUs.

## 4. Discussion

Despite numerous studies on vaccination against *Campylobacter*, there is currently no available vaccine against *Campylobacter* on the market, and most vaccinal approaches are still under development. In fact, the antigens used in such studies have not been effective or have not yielded reproducible results. Moreover, the mechanisms of protection against *Campylobacter* following vaccination are not fully elucidated. In a previous study using a DNA prime/protein boost flagellin-based vaccine, *Campylobacter* spp. was no longer detected (detection limit of the method: 2 log_10_ CFU/g) in 40-day-old SPF White Leghorn chickens challenged with *Campylobacter*. However, this first study did not include an in-depth analysis of the immune mechanism of caecal microbiota accompanying the protection observed [10]. The objective of the present work was to better characterise the immune responses and the microbiota following this flagellin-based vaccination in order to identify factors that could be taken into consideration to improve future vaccines against *Campylobacter* in chickens.

In this present work, and contrary to the previous study, only a partial mean reduction (1.3 log_10_ CFU/g) of *Campylobacter* in the caecal contents of chickens was observed. This different result could be due to the bacterial strain used. In the previous trial, the *C. jejuni* 81–176 strain isolated from humans was used, whereas in this work the *C. jejuni* C97Anses640 strain isolated from poultry was used. The two strains could have a different ability to colonise the chicken caeca, as already suggested [39]. Consequently, the response to flagellin vaccination may be influenced by the strain of *Campylobacter* used, which could explain the difference in protection. Thus, the strength of the immune response against flagellin could have been sufficient to more effectively reduce colonisation by the *C. jejuni* 81–176 strain than the *C. jejuni* C97Anses640 strain.

Moreover, the flagellin used as antigen has already been studied and variable results are suggested [10]. Some studies reported no reduction in *Campylobacter* in broilers [40,41] whereas others reported up to 2 and 3 log_10_ reduction in Leghorn and broilers respectively [42,43]; therefore the reduction of 1.3 log_10_ observed in our study is in agreement with other studies.

In this work, an ELISA was developed to measure the levels of systemic anti-flagellin IgY and mucosal anti-flagellin IgA antibodies. A higher production of systemic anti-flagellin IgY was observed in the vaccinated group than in the placebo group from D19 to D40, as was also previously observed [10] using an ELISA targeting total *Campylobacter* soluble protein (including flagellin). Here too, no correlation could be established between the IgY levels and the level of protection against *Campylobacter*. These observations are in agreement with those of other studies [44].

The production of specific mucosal anti-flagellin IgA antibodies was analysed on several days from D19 to D40, an approach not applied in the previous study (when these antibodies were only examined at slaughter on D42). Higher levels of IgA were observed from D22 to D40 in the vaccinated group than in the placebo one, whereas the opposite was found when measured on D42 in the previous study [10]. Several hypotheses could explain these reverse results: (1) due to the low level of *Campylobacter* in the caeca in the previous study, the mucosal immune response may not have been stimulated in the vaccinated group (2) or IgA could have been produced and consumed to fight *Campylobacter* [10]. Moreover, IgA have a short half-life [45], so they could not have been detected at the time of the day 42 analysis, when no *Campylobacter* were detected. Even though it has not been formally demonstrated, anti-*Campylobacter* IgA may be involved to a certain extent in the protection against *Camplylobacter*. However, as already suggested in the previous study [10], no correlation between the IgA levels and the level of protection against *Campylobacter* could be established. Thus, as already suggested, the protection observed may not be strictly antibody-mediated [44]. These results showed that vaccination primed the immune response but discrimination between the responses due to the vaccination alone or both vaccination and *Campylobacter* colonisation is not possible.

In response to *Campylobacter* colonisation, the relative expression of several chemokines, cytokines and AMPs reflecting the activation of different innate and specific immune pathways have been demonstrated. Antimicrobial peptides such as AvBD10 and AvBD12 involved in the innate immune pathways, were upregulated in the presence of *Campylobacter* [20,46]. Furthermore, activation of the Th1 pathway with the pro-inflammatory IFN-γ [16,47] and Th2 pathway with IL-4 [16] were upregulated in response to *Campylobacter*. Activation of the Th17 pathway against *Campylobacter* was also demonstrated with an upregulation of pro-inflammatory targets such as IL-1β, IL-17A, IL8L1 and IL8L2 that could play a role in protecting against *Campylobacter* [16,19,48]. Moreover, the anti-inflammatory IL-10 involved in the Treg pathway was upregulated in response to *Campylobacter* according to several studies, no doubt to decrease inflammatory damage [16,47].

To the best of our knowledge, the panel of targets used in the present study had not previously been evaluated in response to vaccination against *Campylobacter*. An increase in IL-10 relative expression was detected on D28 in the vaccinated group, compared with the placebo one. This cytokine is produced by regulatory T (Treg) cells, known to have an anti-inflammatory role to minimise tissue damage. IL-10-mediated B cell regulatory activity leads to a reduction in T cell functions [49]. However, no significant relative over-expression was measured for any of the other eight targets corresponding to cytokines and chemokines representing cellular immune responses and antimicrobial peptides. This suggests that such responses may not be stimulated by this vaccination, which resulted in only partial protection.

An over-expression of AvBD10 in the placebo group compared with the vaccinated group was shown on day 40. The upregulation of β-defensins has previously been described in response to *Campylobacter* colonisation, denoting the importance of the role of β-defensins as part of the local intestinal host response [20,46]. An upregulation of AvBD10, characterising an innate immune response, could be an attempt by the host to prevent or limit the bacteria’s colonisation. Future research should investigate cells producing cytokines and chemokines to bring new insights on their role in response to *Campylobacter* vaccination.

This study clearly demonstrated modifications to the composition and structure of chicken caecal microbiota due to the vaccination from D19, i.e., before the *Campylobacter* challenge, and more specifically, through to the end of the trial on D40 in the group receiving the flagellin-based vaccination, as the vaccination explained 80% of the total variance. Another study investigating the effects of a flagellin-like protein without a *Campylobacter* challenge also reported a modulated caecal microbiota among SPF Leghorn chickens in response to the protein [50]. A modulation of the caecal microbiota was also observed in response to oral vaccination against *Campylobacter* in commercial broilers [26]. On the contrary, another study showed that immunising SPF White Leghorn chickens with an enterobactin conjugate vaccine induced a strong level of systemic IgY and a significant reduction in *C. jejuni* colonisation of 3–4 log_10_ CFU/g of caecal content, but no significant difference was observed in the caecal microbiota [11].

In this work, the vaccinated group demonstrated a slight but significant reduction in *Campylobacter* counts in the caeca; this reduction was confirmed by an analysis of the microbiota, as the genus *Campylobacter* was differentially associated with the placebo group. A higher abundance of *Firmicutes*, *Ruminococcaceae*, *Clostridiales*, *Blautia* and *Faecalibacterium* was also observed in the vaccinated group. Previous works have tried to identify taxa that could be associated with the presence of *Campylobacter* in poultry, but the results are conflicting. For example, one study suggested that *Faecalibacterium* interacted with *Campylobacter* [25,26], but this result was not confirmed in another study [51]. Moreover, an increase in *Blautia* and *Clostridiales* was associated with *Campylobacter* [19] while another study reported a decrease in *Blautia* but an increase in *Clostridiales* [26]. In the light of such results, it is not possible to conclude whether the modifications observed in the vaccinated group may be associated with the reduction in *Campylobacter*. This work suggests that vaccination could impact microbiota, but chickens SPF have a limited resident gut microbiota unlike conventional chickens. For example, *Bacteroidetes* phylum is one of the major phyla in conventional chicken but was not identified in SPF chickens in this work. Effect of vaccination on broiler caecal microbiota could be further studied.

The microbiota and the immune system co-evolve in the chicken gut, and their balanced relationship is based on crosstalk throughout the chicken’s life [52]. Interactions between the immune system and the gut microbiota of chickens challenged with *C. jejuni* are starting to be described in the literature [17,19]. The present work demonstrated that IgY and IgA antibodies were produced, and the microbiota modified in response to vaccination, but only a slight reduction in *Campylobacter* was observed. Indeed, it was not possible to conclude whether these parameters could act individually or interact together to reduce *Campylobacter* colonisation or if they are not involved. Further studies are needed to better understand the role of these parameters on *Campylobacter* colonisation. However, it is important to mention that other issues need to be overcome in order to develop effective vaccines against *Campylobacter.* These include the short life span of broilers and consequently the young and immunologically naïve host chicken [18]. Moreover, *Campylobacter* is considered as non-pathogenic in the intestinal tract of poultry hosts [28]. Thus, chickens first react as if colonisation by *C. jejuni* is an attack until they reach a certain level of tolerance [53].

## 5. Conclusions

This study demonstrated that vaccination resulted in a slight reduction in the caecal *Campylobacter* load in chickens. This flagellin-based vaccine strongly stimulated the systemic and mucosal humoral pathway and affected the structure and composition of the caecal microbiota, but the link between these modifications and the *Campylobacter* reduction remains to be elucidated. These new data demonstrate that both immune responses and microbiota composition need to be explored in order to clarify their role against *Campylobacter* in poultry and thus improve future vaccination protocols.

## Figures and Tables

**Table 1:** Primer sequences for the gene expression determined by qPCR.

Target Gene	Primer Sequence (5′-3′)	Product Size (bp)	NCBI Accession Number	Reference
B-actin	F: CCCACCTGAGCGCAAGTACT R: AAGCATTTGCGGTGGACAAT	132	NM_205518.1	[31]
IFN-γ	F: TGAGCCAGATTGTTTCGATG R: CTTGGCCAGGTCCATGATA	152	NM_205149.1	[32]
IL-1β	F: GTGAGGCTCAACATTGCGCTGTA R: TGTCCAGGCGGTAGAAGATGAAG	214	NM_204524.1	[32]
IL-4	F: GCTCTCAGTGCCGCTGATG R: GAAACCTCTCCCTGGATGTCAT	60	NM_204524.1	[33]
IL-10	F: CGCTGTCACCGCTTCTTCA R: CGAACGTCTCCTTGATCTGCTT	67	NM_001004414.2	Primer Express^®^
IL-17A	R: CATGGGATTACAGGATCGATGA F: GCGGCACTGGGCATCA	68	NM_204460.1	[16]
IL8L1	F: CCGATGCCAGTGCATAGAG R: CCTTGTCCAGAATTGCCTTG	191	NM_205018.1	[34]
IL8L2	F: CCTGGTTTCAGCTGCTCTGT R: GCGTCAGCTTCACATCTTGA	128	NM_205498.1	[34]
AvBD10	F: CAGACCCACTTTTCCCTGACA R: CCCAGCACGGCAGAAATT	64	NM_001001609.2	[35]
AvBD12	F: TGTAACCACGACAGGGGATTG R: GGGAGTTGGTGACAGAGGTTT	114	NM_001001607.2	[35]

**Table 2:** Body weights (mean ± SD in g) of chicken groups during the trial.

Groups	Day 19	Day 22	Day 28	Day 35	Day 40
Placebo	181 ± 27	218 ± 37	312 ± 40	432 ± 53	524 ± 74
Vaccinated	183 ± 22	223 ± 30	319 ± 31	443 ± 48	530 ± 58

**Table 3:** Number of sequences and OTUs per step. Ten chickens (five in the vaccinated group and five in the placebo group) on D19 and D40 and eight chickens (four in the vaccinated group and four in the placebo group) on D22 were tested.

	D19	D22	D40
Number of sequences after read demultiplexing and pre-process (merging, denoising and dereplication)	1,133,635 (min: 74,225; max: 153,026)	735,454 (min: 61, 456; max: 125,181)	1,032,938 (min: 45,196; max: 163,639)
Number of OTU	332 Placebo: (min: 219; max:263) Vaccinated: (min: 163, max:234)	348 Placebo: (min: 256; max: 299) Vaccinated: (min: 115, max: 182)	412 Placebo: (min: 203; max: 296) Vaccinated: (min: 271, max: 314)

## Data Availability

Not applicable.

## Supplementary Materials

The following supporting information can be downloaded from https://www.mdpi.com/article/10.3390/vaccines10060981/s1, Table S1: Alpha diversity index values for all the chicken caecal samples; Table S2: Table showing relative abundance of OTUs and their taxonomic affiliation in chicken caecal samples on day 40.
